# Comparison of the prognostic value of a comprehensive set of predictors in identifying risk of metabolic-associated fatty liver disease among employed adults

**DOI:** 10.1186/s12889-023-15365-9

**Published:** 2023-03-29

**Authors:** Ze Yang, Bin Yu, Zihang Wang, Zhitao Li, Bo Yang, Honglian Zeng, Shujuan Yang

**Affiliations:** 1 Social Insurance Administration Department, China Railway Chengdu Group Company, Ltd, Chengdu, China; 2 Institute for Disaster Management and Reconstruction, Sichuan University, Chengdu, China; 3 West China School of Public Health and West China Fourth Hospital, Sichuan University, Chengdu, Sichuan China; 4 Affiliated Hospital of Chengdu University, Chengdu, Sichuan China; 5 International Institute of Spatial Lifecourse Health (ISLE), Wuhan University, Wuhan, China

**Keywords:** Metabolic-associated fatty liver disease, Employed adults, Prediction performance, Triglyceride glucose-body mass index

## Abstract

**Objective:**

Metabolic-associated fatty liver disease (MAFLD) is of concern in employed adults, while the crucial indicators in predicting MAFLD are understudied in this population. We aimed to investigate and compare the prediction performance of a set of indicators for MAFLD in employed adults.

**Methods:**

A cross-sectional study recruiting 7968 employed adults was conducted in southwest China. MAFLD was assessed by abdominal ultrasonography and physical examination. Comprehensive indicators of demographics, anthropometric, lifestyle, psychological, and biochemical indicators were collected by questionnaire or physical examination. All indicators were evaluated for importance in predicting MAFLD by random forest. A prognostic model based on multivariate regression model was constructed to obtain a prognostic index. All indicators and prognostic index were compared to evaluate their prediction performance in predicting MAFLD by the receiver operating characteristic (ROC) curve, calibration plot, and Decision curve analysis (DCA).

**Results:**

Triglyceride Glucose-Body Mass Index (TyG-BMI), BMI, TyG, triglyceride (TG)/high-density lipoprotein-cholesterol (HDL-C), and TG ranked the top five important indicators, and TyG-BMI performed the most accurate prediction of MAFLD according to the ROC curve, calibration plot and DCA. The area under the ROC curves (AUCs) of the five indicators were all over 0.7, with TyG-BMI (cut-off value: 218.284, sensitivity: 81.7%, specificity: 78.3%) suggesting the most sensitive and specific indicator. All five indicators showed higher prediction performance and net benefit than the prognostic model.

**Conclusion:**

This epidemiological study firstly compared a set of indicators to evaluate their prediction performance in predicting MAFLD risk among employed adults. Intervention targeting powerful predictors can be helpful to reduce the MAFLD risk among employed adults.

**Supplementary Information:**

The online version contains supplementary material available at 10.1186/s12889-023-15365-9.

## Introduction

Metabolic-associated fatty liver disease (MAFLD), a disease renamed from non-alcoholic fatty liver disease (NAFLD), is currently the most common cause of chronic liver disease globally [1]. It is reported that the prevalence of MAFLD in Asia is ranging from 15% to 40%, and this rate still has a continuous growth trend, posing a substantial economic burden to society [2]. For some high-risk populations such as employed adults with frequently irregular lifestyles and shift work [3, 4], the MAFLD is of concern. As is reported, the prevalence of MAFLD in employed adults is around 40% in China [5–7]. Stacks of evidence suggested that MAFLD is closely associated with poor health consequences, such as cirrhosis and hepatocellular carcinoma [8, 9]. Early identification of the prognosis of MAFLD among employed adults contributes to early interventions before the irreversible health consequence.

To strangle chronic liver disease in its cradle, many indicators for early detection and prevention of MAFLD have been studied. For example, anthropometric characteristics (e.g., Body Mass Index [BMI], waistline) are proposed as important factors for the development of MAFLD [10]; lifestyles (e.g., smoking [11], drinking [12], dietary pattern [13]), which are generally known modified factors for chronic disease development, were also found closely associated with MAFLD. In addition to these aforementioned factors, MAFLD is particularly determined by blood biochemical indicators. Under the “multi-hit” pathogenesis theory of MAFLD, insulin resistance (IR) [14] is crucial to developing MAFLD, while the measurement of IR is time-consuming and expensive. Triglyceride to high-density lipoprotein cholesterol (TG/HDL-C) and triglyceride and glucose index (TyG), as the predictors and substitutes of IR, have been suggested as useful, cheap, and convenient biomarkers in predicting MAFLD [15]. TyG-BMI is a reliable substitute for TyG in predicting MAFLD as proposed by Ko et al. in 2016 [16]. One previous study among 229 hospitalized patients showed that TyG-BMI revealed a good prediction performance in the diagnosis of MAFLD [17]. Other biomarkers such as TG, serum alanine transaminase (ALT) also take important roles in predicting MAFLD [18]. Psychological factors such as perceived stress might affect MAFLD [19], though related evidence is insufficient. Up until now, studies have evaluated prediction performance of a specific index (e.g., TyG-BMI) [20, 21] or compared the predictive performance of a set of indicators for MAFLD among general population [22]. However, few studies compared prediction performance of these indicators in predicting MAFLD among employed adults.

This study, based on a large sample of employed adults in China, aimed to compare the prediction performance of a set of demographics, anthropometric, lifestyle, psychological, and blood biochemical indicators for MAFLD among employed adults. Our findings could help understand the indicators’ ability to predict MAFLD risk and develop early interventions targeting powerful predictors to reduce the MAFLD risk and poor prognosis among employed adults.

## Methods

### Study design and data collection

This cross-sectional study was conducted in southwest China from January to December 2021. Participants were recruited from the China Railway Chengdu Group Company by posting a notice on the enterprise personnel management website. A total of 8,520 participants from 50 railway stations in Sichuan Province, Guizhou Province, and Chongqing municipality who underwent physical examinations in 2021 voluntarily participated in the study.

Participants inspected fatty liver with abdominal ultrasonography, and the reports were obtained from the Social Insurance Administration Department of China Railway Chengdu Group Company. Fasting blood samples (e.g., glucose and lipids) were collected after at least an 8-h overnight fast. All blood samples were sent to the lab of Clinical Medical College & Affiliated Hospital of Chengdu University for analysis. Participants were asked to wear light clothing and barefoot to measure their weight, height, and waist circumference. The BMI was calculated as weight (kg)/height (m) ^2^. Besides, the blood pressure of each participant was measured three times at a resting state. When physical examination data were collected, face-to-face questionnaire surveys were performed at the same time for each participant to collect their demographics, lifestyle, and psychological information. All physical examinations and questionnaire surveys were conducted by trained medical workers in the affiliated hospital of Chengdu University. A total of 110 participants with any incomplete questionnaire or physical examination data (except variables used to define MAFLD) were excluded. Besides, we excluded 343 patients with hepatitis B or hepatitis C, 47 patients with alcoholic liver diseases, and 158 people who used medication due to these liver diseases. Finally, a total of 7,968 participants were included in this study. The ethical approval of this study was received from the Ethics Committee of the Affiliated Hospital of Chengdu University (PJ 2019–015-02).

### Measurement of MAFLD

MAFLD was defined as fatty liver with overweight (Body Mass Index [BMI] ≥ 23 kg/m^2^) or type 2 diabetes mellitus [23], or at least two metabolic risk abnormalities as follows: 1) waist circumference ≥ 90 for male and ≥ 80 cm for female; 2) blood pressure ≥ 130/85 mmHg; 3) plasma triglycerides ≥ 1.70 mmol/L; 4) plasma HDL-C < 1.0 for male and < 1.3 mmol/L for female; 5) fasting glucose levels 5.6 to 6.9 mmol/L, or 2-h post-load glucose levels 7.8 to 11.0 mmol/L, or HbA1c 5.7% to 6.4%; 6) homeostasis assessment of IR score ≥ 2.5; and 7) plasma high-sensitivity C-reactive protein level > 2 mg/L [24]. In the current studies, only six criteria were considered since absent information on plasma insulin levels. 

### Candidate predictors of MAFLD

Referring to the previous studies on the predictive biomarkers for MAFLD [17, 25], we contained a set of biochemical indicators as candidate predictors, including total bilirubin (TBIL), direct bilirubin (DBIL), indirect bilirubin (IBIL), ALT, aspartate transaminase (AST), total protein (TP), albumin (ALB), globulin (GLB), fasting plasma glucose (FPG), uric acid (UA), urea (UREA), creatinine (CREA), TG, total cholesterol (TC), HDL, and low-density lipoprotein cholesterol (LDL). Besides, we calculated a series of composite indicators, including TyG, ALT/AST, LDL-C/HDL-C, TG/HDL-C, TC/HDL, TyG-BMI, and Systemic immune-inflammation index (SII). The TyG index was calculated as Ln [(fasting TG (mg/dl)*FBG (mg/dl)/2] [26]. TyG-BMI are defined as TyG index*BMI [27]. SII was a novel index developed based on lymphocyte, neutrophil, and platelet counts, and has been proved as a powerful prognostic indicator of poor outcome in patients with hepatocellular carcinoma [28]. SII was calculated as total peripheral platelets count (P)*neutrophil-to-lymphocyte (N/L) ratio (SII = P*N/L ratio) [28]

Additionally, we included a series of demographics, anthropometric, lifestyle and psychological factors as candidate predictors of MAFLD, including 1) demographics and anthropometrics, i.e., sex (male, female), age, ethnicity (Han, minority), educational level (high school and below, undergraduate or above), marital status (married/cohabitation, unmarried, separated/divorced/widowed), monthly household income (< 6,000 *yuan*, ≥ 6,000 *yuan*), living status (living with family members, colleagues/friends, strangers, or alone), and BMI; 2) lifestyle and psychological factors, i.e., drinking (no [non-drinker], yes [ex-drinker, occasional drinker, current drinker]), smoking (no [non-smoker], yes [ex-smoker, occasional smoker, current smoker]); dietary pattern was evaluated by the food frequency questionnaire (FFQ) scale, which can be divided into three groups of ideal, medium, and poor dietary pattern [29, 30]; physical activities was measured by the Metabolic Equivalent of Task (MET) [31], and divided into < 600 MET-min/w, 600–2999 MET-min/w, and ≥ 3000 MET-min/w; sleep quality was estimated by the Pittsburgh Sleep Quality Index (PSQI) [32] with a total score ranging from 0 to 21, and PSQI total score ≤ 5 was defined as good sleep quality; perceived stress was measured by the stress mindset measurement [33], and a high score indicating a high a level of stress perception.

### Statistical methods

Descriptive analysis was conducted, with mean ± standard deviation (SD) for continuous variables and proportion (%) for categorical variables. Comparison of the candidate predictors between participants with and without MAFLD was performed by t-test (for continuous variables) or chi-square tests (for categorical variables). Among 7,968 participants, 2,669 were with missing data of waist circumference and 5 were with missing data of other variables used to define MAFLD. Therefore, multiple imputations with chained equations on 25 sets was used for missing data before data analysis [34].

A variable importance plot based on the random forest was employed to rank the importance of candidate predictors in explaining the variance of dependent variables [35, 36], and we considered the top five important indicators to compare their prediction performance for MAFLD. Multivariate analysis with binary logistic regression models was used to identify the indicators significantly associated with MAFLD, with association estimated by odds ratio (OR) and 95% confidence interval (CI). To strictly control the collinearity of variables, we used the variance inflation factor (VIF) controlled below 2.5 to obtain the predictors statistically significant in the prognostic model. The Nomograms were used to visualize the results of the prognostic model and obtain the prognostic index for MAFLD [37]. Since we aimed to compare the prediction performance of top five indicator with the prognostic index, we therefore excluded the five indicators as candidate predictors of the prognostic model. By this way we can understand whether these individual predictors predict better than the synthetic composite indicator based on remaining indicators and avoid potential collinearity between these predictors.

The receiver operating characteristic (ROC) curve with the area under the ROC curve (AUC) was used to test the prediction performance of each predictor for MAFLD risk [38], with 0.5 and 1.0 indicating no and complete predictive ability, respectively, and AUC > 0.7 was considered as acceptable prediction performance. The maximum value of the Youden index (i.e., sensitivity + specificity-1) was taken as the optimal cut-off value [39]. The calibration curves were used to illustrate the prediction ability of the Nomogram by comparing the predicted and the observed probabilities of MAFLD after bias correction. Decision curve analysis (DCA) was also used to determine and compare the net clinical benefits by the predictors [40]. To evaluate the reproducibility of the findings on prediction performance, participants in the total sample were randomly allocated to a training set (70%) and a validation set (30%) and repeated above depicted process [41]. Besides, we conducted all analysis in participants without missing data, i.e., a sample excluding participants with any incomplete questionnaire or physical examination data, to test the replicability of the findings.

R software (version 4.1.1) was used for all statistical analyses, and the statistical significance levels were set at 0.05.

## Results

### Baseline characteristics

The demographics, lifestyles-related and psychological characteristics, and biomarkers of participants were presented in Table 1. The mean age of the 7,968 participants was 39.6 ± 11.0 years, and 93.1% (*n* = 7,415/7,968) of them were men. The prevalence of MAFLD was as high as 41.0% (*n* = 3,268/7,968). Compared with those with non-MAFLD, those with MAFLD tend to be male, older, less educational, married, with a higher household income, working indoors, living with family members, with a higher BMI, current drinkers, and current smokers (Table 1).

**Table 1:** The basic characteristics of the participants

Variable	Total	MAFLD (n[%] or mean ± SD)		
		No	Yes	*P* value
	*N* = 7,968	*N* = 4,700	*N* = 3,268	
Sex				< 0.001
Male	7,415 (93.06)	4,220 (89.79)	3,195 (97.77)	
Female	553 (6.94)	480 (10.21)	73 (2.23)	
Age (years)	39.60 ± 10.96	37.79 ± 11.00	42.21 ± 10.37	< 0.001
Ethnicity				0.494
Han	7,518 (94.35)	4,442 (94.51)	3,076 (94.12)	
Minority	450 (5.65)	258 (5.49)	192 (5.88)	
Educational level				< 0.001
High school or below	3,095 (38.84)	1,557 (33.13)	1,538 (47.06)	
Undergraduate or above	4,873 (61.16)	3,143 (66.87)	1,730 (52.94)	
Marital status				< 0.001
Married/cohabitation	5,667 (71.12)	3,111 (66.19)	2,556 (78.21)	
Unmarried	1,904 (23.90)	1,368 (29.11)	536 (16.40)	
Separated/Divorced/Widowed	397 (4.98)	221 (4.70)	176 (5.39)	
Monthly household income (*yuan*)				0.001
< 6,000	2,347 (29.46)	1,454 (30.94)	893 (27.33)	
≥ 6,000	5,621 (70.54)	3,246 (69.06)	2,375 (72.67)	
Work type				0.303
Work outdoor	2,147 (26.95)	1,287 (27.38)	860 (26.32)	
Work indoor	5,821 (73.05)	3,413 (72.62)	2,408 (73.68)	
Living status				< 0.001
Living with family members	5,189 (65.12)	2,893 (61.55)	2,296 (70.26)	
Living with colleagues or friends	1,177 (14.77)	799 (17.00)	378 (11.57)	
Living with strangers	216 (2.71)	133 (2.83)	83 (2.54)	
Living alone	1,386 (17.39)	875 (18.62)	511 (15.64)	
BMI (kg/m^2^)	24.41 ± 3.52	22.73 ± 2.74	26.83 ± 3.08	< 0.001
Drinking				< 0.001
Non-drinker	2,901 (36.41)	1,842 (39.19)	1,059 (32.41)	
Ex-drinker	359 (4.51)	198 (4.21)	161 (4.93)	
Occasional drinker	3,225 (40.47)	1,922 (40.89)	1,303 (39.87)	
Current drinker	1,483 (18.61)	738 (15.70)	745 (22.80)	
Smoking				< 0.001
Non-smoker	3,037 (38.11)	2,022 (43.02)	1,015 (31.06)	
Ex-smoker	583 (7.32)	292 (6.21)	291 (8.90)	
Occasional smoker	890 (11.17)	539 (11.47)	351 (10.74)	
Current smoker	3,458 (43.40)	1,847 (39.30)	1,611 (49.30)	
Dietary pattern				0.824
Ideal dietary pattern	2,855 (35.83)	1,697 (36.11)	1,158 (35.43)	
Medium dietary pattern	4,766 (59.81)	2,800 (59.57)	1,966 (60.16)	
Poor dietary pattern	347 (4.35)	203 (4.32)	144 (4.41)	
Physical activity (MET-min/w)				0.594
< 600	2,552 (32.03)	1,521 (32.36)	1,031 (31.55)	
600–2999	2,734 (34.31)	1,617 (34.40)	1,117 (34.18)	
≥ 3,000	2,682 (33.66)	1,562 (33.23)	1,120 (34.27)	
Sleep quality				0.385
Good	4,146 (52.03)	2,426 (51.62)	1,720 (52.63)	
Poor	3,822 (47.97)	2,274 (48.38)	1,548 (47.37)	
Stress perception	16.33 ± 3.81	16.29 ± 3.85	16.38 ± 3.77	0.282
TBIL (μmold/L)	16.39 ± 6.16	16.69 ± 6.26	15.95 ± 6.00	< 0.001
DBIL (μmold/L)	5.33 ± 2.30	5.49 ± 2.35	5.09 ± 2.21	< 0.001
IBIL (μmold/L)	11.06 ± 4.18	11.20 ± 4.21	10.86 ± 4.13	< 0.001
ALT (IU/L)	23.53 ± 12.12	21.32 ± 9.85	26.71 ± 14.19	< 0.001
AST (IU/L)	29.94 ± 24.19	22.80 ± 17.23	40.19 ± 28.65	< 0.001
TP (g/L)	75.38 ± 4.41	75.07 ± 3.98	75.83 ± 4.94	< 0.001
ALB (g/L)	45.22 ± 2.36	45.24 ± 2.37	45.20 ± 2.34	0.463
GLB (g/L)	30.16 ± 3.79	29.83 ± 3.32	30.63 ± 4.33	< 0.001
FPG (mmol/L)	5.45 ± 1.32	5.17 ± 0.88	5.84 ± 1.69	< 0.001
UA (μmold/L)	396.31 ± 87.68	376.00 ± 81.56	425.51 ± 87.94	< 0.001
UREA (mmol/L)	4.87 ± 1.20	4.84 ± 1.20	4.91 ± 1.20	0.009
CREA (mmol/L)	71.73 ± 12.19	71.19 ± 11.96	72.51 ± 12.46	< 0.001
TG (mmol/L)	2.05 ± 1.87	1.53 ± 1.22	2.79 ± 2.34	< 0.001
TC (mmol/L)	4.69 ± 0.90	4.52 ± 0.85	4.92 ± 0.93	< 0.001
HDL-C (mmol/L)	1.37 ± 0.31	1.45 ± 0.31	1.25 ± 0.27	< 0.001
LDL-C (mmol/L)	2.71 ± 0.68	2.56 ± 0.65	2.94 ± 0.66	< 0.001
TyG	8.86 ± 0.68	8.58 ± 0.55	9.25 ± 0.66	< 0.001
ALT/AST	0.97 ± 0.46	1.10 ± 0.49	0.77 ± 0.30	< 0.001
LDL-C/HDL-C	2.10 ± 0.88	1.85 ± 0.64	2.46 ± 1.04	< 0.001
TG/HDL-C	1.84 ± 4.49	1.21 ± 1.86	2.74 ± 6.54	< 0.001
TC/HDL-C	3.60 ± 1.45	3.24 ± 0.92	4.12 ± 1.85	< 0.001
TyG-BMI	217.15 ± 41.04	195.58 ± 30.00	248.17 ± 34.38	< 0.001
SII	425.32 ± 212.24	418.20 ± 216.15	435.57 ± 206.09	< 0.001

*MAFLD* Metabolic-associated fatty liver disease, *BMI* Body Mass Index, *MET* Metabolic Equivalent of Task, *TBIL* Total bilirubin, *DBIL* Direct bilirubin, *IBIL* Indirect bilirubin, *ALT* Serum alanine transaminase, *AST* Aspartate transaminase, *TP* Total protein, *ALB* Albumin, *GLB* Globulin, *FPG* Fasting plasma glucose, *UA* Uric acid, *UREA* Urea, *CREA* Creatinine, *TG* Triglycerides, *TC* Total cholesterol, *HDL-C* High-density lipoprotein cholesterol, *LDL-C* Low-density lipoprotein cholesterol, *TyG* Triglyceride and glucose index, *SII* Systemic immune-inflammation index, *SD* Standard deviation

### Important predictor selection

**Fig. 1:**
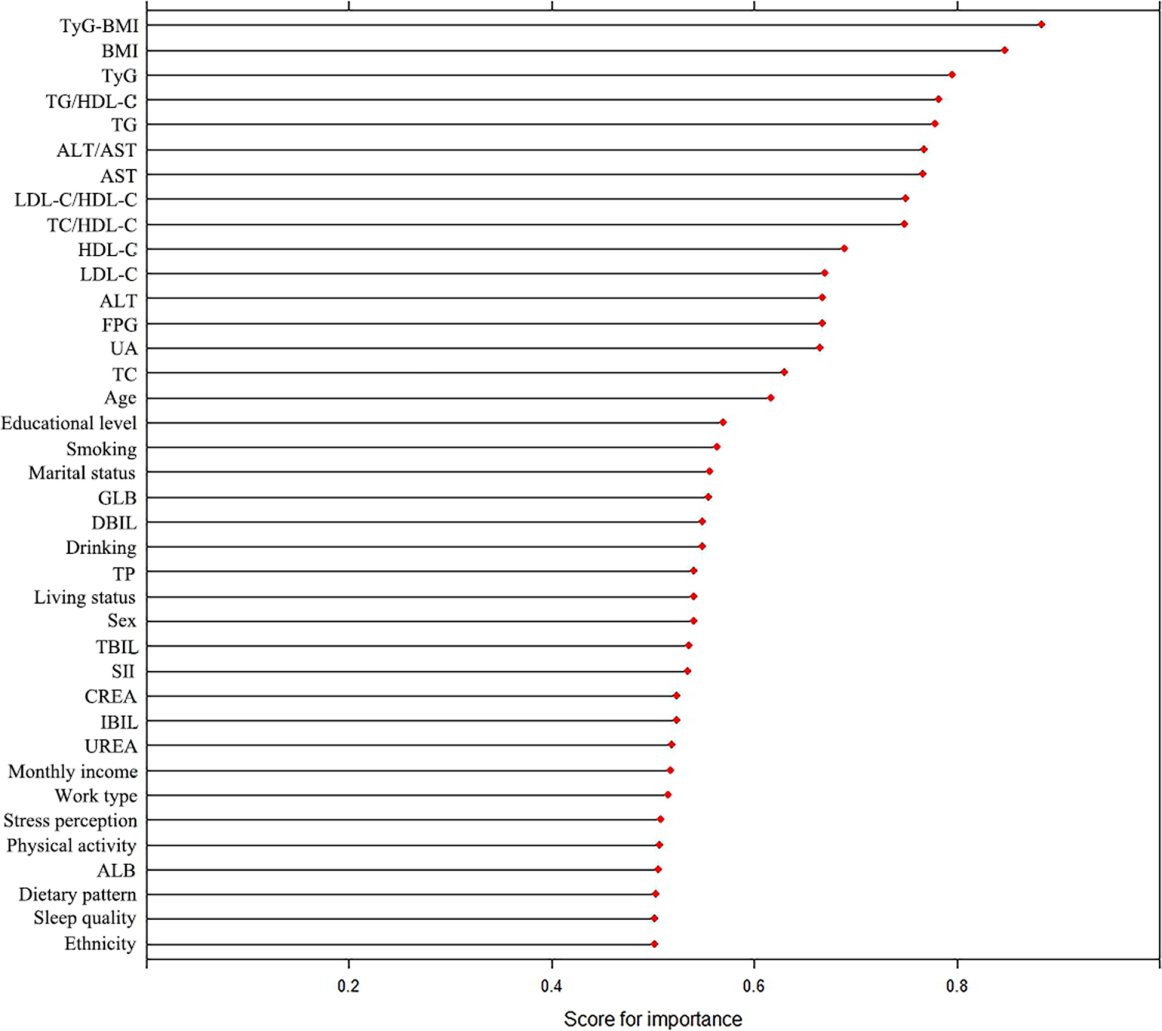
Importance score of candidate predictors. Note: variable importance suggested the difference in prediction error when feature values are altered randomly to the prediction error, with a higher difference correspondence to a higher importance of the feature. Abbreviation: TyG: triglyceride and glucose index; BMI: Body Mass Index; TG: triglycerides; HDL-C: high-density lipoprotein cholesterol; ALT: serum alanine transaminase; AST: aspartate transaminase; LDL-C: low-density lipoprotein cholesterol; FPG: fasting plasma glucose; UA: uric acid; TC: total cholesterol; GLB: globulin; DBIL: direct bilirubin; TP: total protein; TBIL: total bilirubin; SII: Systemic immune-inflammation index; CREA: creatinine; IBIL: indirect bilirubin; UREA: urea; ALB: albumin

Among all indicators, by using the random forest method, we found biochemical indicators were generally more important than demographic, lifestyles, and psychological indicators in explaining the prevalence of MAFLD. Specifically, TyG-BMI, BMI, TyG, TG/HDL-C, and TG ranked top five important predictors (Fig. 1).

**Fig. 2:**
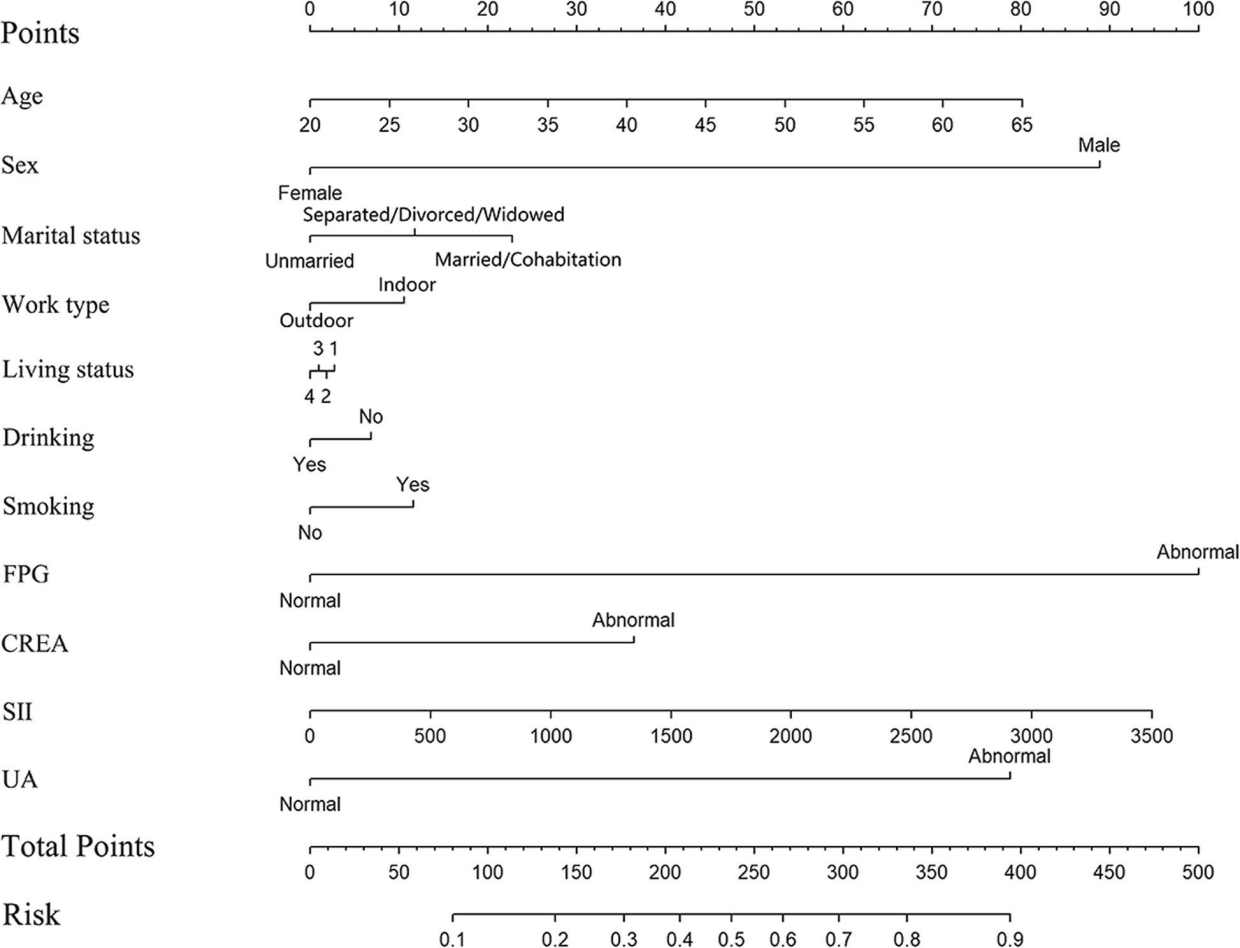
Nomogram of the diagnostic model. Note: Drawing a vertical line from the axis of each predictor until it reaches the line labeled by “points”, we can calculate the point of each predictor. The summed score of points of all predictors was the total points. Drawing a vertical line in the line labeled “Total Points” from the obtained value of total points until it intercepts the line labeled “Risk”, we can obtain the risk (predicted probability) of MAFLD for a given individual. Abbreviation: MAFLD: Metabolic-associated fatty liver disease; FPG: fasting plasma glucose; CREA: creatinine; SII: Systemic immune-inflammation index; UA: uric acid. 1: living with family member, 2: living with strangers, 3: living alone, 4: living with colleagues or friends

To understand whether a synthetic composite indicator based on remaining indicators still suggest a better prediction performance than the five individual indicators, we developed a Nomogram including indicators significantly associated with MAFLD and were with VIF below 2.5. Ultimately, a Nomogram based on eleven predictors (i.e., age, sex, marital status, work type, living status, drinking, smoking, FPG, CREA, SII, and UA) was obtained (Fig. 2). The probability of diagnosed MAFLD was obtained, and the prognostic index was calculated by 1.778*(Age-20)+88.328*Sex+11.368*Marital status+10.571*Work type+1.442*Living status+6.512*Drinking+11.902*Smoking+100.000*FPG+36.992*CREA+0.026*SII+78.731*UA.

### Comparison of prediction performance

**Fig. 3:**
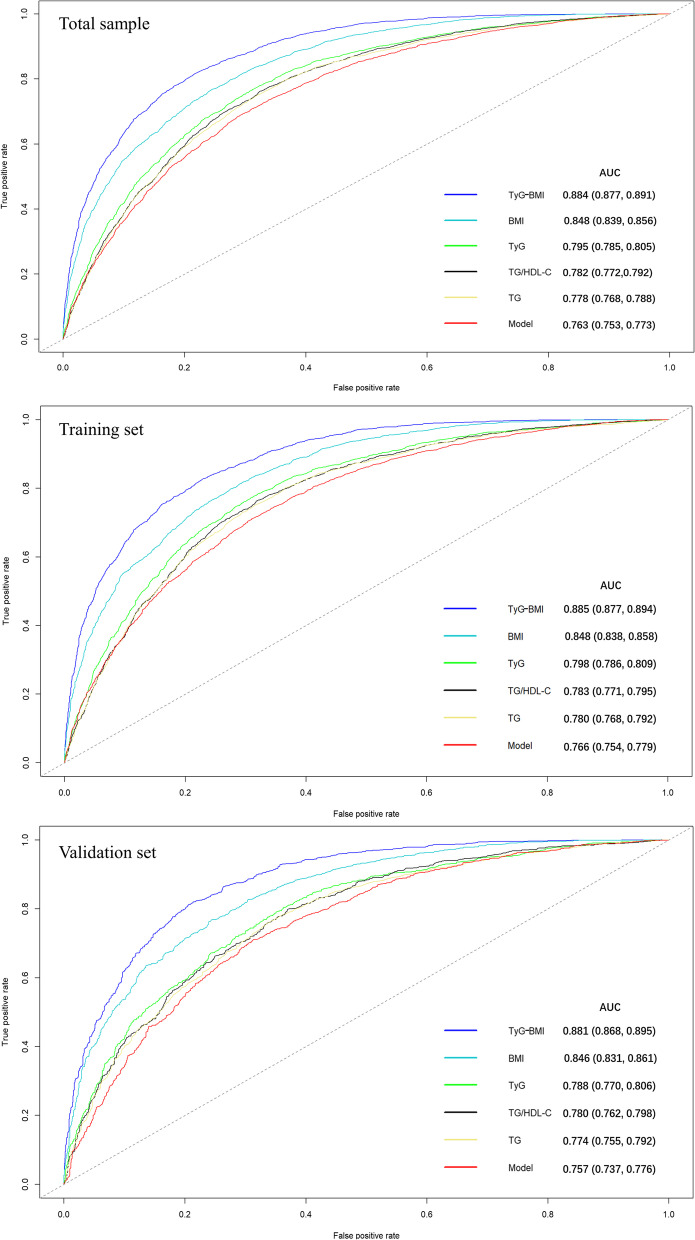
ROC curves of TyG-BMI, BMI, TyG, TyG/HDL-C, TG, and the Model for MAFLD. Note: Model: prognostic model. Abbreviation: MAFLD: Metabolic-associated fatty liver disease; TyG: triglyceride and glucose index; BMI: Body Mass Index; HDL: high-density lipoprotein cholesterol; TG: triglycerides

A ROC curve was developed to compare the five indicators and the prognostic index in the prediction performance for MAFLD. As shown in Fig. 3, the TyG-BMI showed the highest AUC (0.884), followed by BMI (0.848), and TyG (0.795). The AUCs of five selected indicators were all over 0.7 and were higher than prognostic index based on remaining indicators, indicating a relatively accurate diagnostic value. For TyG-BMI and BMI, the values of sensitivity and specificity indices were over 0.7, and the TyG-BMI showed the most accurate predictor of MAFLD (cut-off value: 218.284, sensitivity: 81.7%, specificity: 78.3%). The specific values of AUC, and sensitivity and specificity for each indicator can be found in Table 2.

**Table 2:** AUC of TyG-BMI, BMI, TyG, TyG/HDL-C, TG, and the Model in predicting MAFLD risk

Variable	AUC	95%CI	Cut-off value	Sensitivity (%)	Specificity (%)	Youden's index
*Total sample*						
TyG-BMI	0.884	(0.877, 0.891)	218.284	0.817	0.783	0.599
BMI	0.848	(0.839, 0.856)	24.615	0.763	0.760	0.523
TyG	0.795	(0.785, 0.805)	8.771	0.779	0.677	0.456
TG/HDL-C	0.782	(0.772,0.792)	1.146	0.762	0.676	0.438
TG	0.778	(0.768, 0.788)	1.475	0.797	0.635	0.432
Model	0.763	(0.753, 0.773)	-0.478	0.736	0.661	0.397
*Training sample*						
TyG-BMI	0.885	(0.877, 0.894)	217.401	0.827	0.772	0.599
BMI	0.848	(0.838, 0.858)	24.335	0.798	0.725	0.524
TyG	0.798	(0.786, 0.809)	8.788	0.772	0.692	0.464
TG/HDL-C	0.783	(0.771, 0.795)	1.150	0.764	0.682	0.446
TG	0.780	(0.768, 0.792)	1.475	0.802	0.634	0.436
Model	0.766	(0.754, 0.779)	-0.426	0.712	0.690	0.401
*Validation sample*						
TyG-BMI	0.881	(0.868, 0.895)	219.406	0.813	0.794	0.607
BMI	0.846	(0.831, 0.861)	24.555	0.768	0.756	0.524
TyG	0.788	(0.770, 0.806)	8.713	0.804	0.638	0.442
TG/HDL-C	0.780	(0.762, 0.798)	1.054	0.800	0.629	0.429
TG	0.774	(0.755, 0.792)	1.525	0.766	0.657	0.423
Model	0.757	(0.737, 0.776)	-0.400	0.710	0.688	0.398

*Abbreviation: AUC* Area under the receiver operating characteristic curve, *Model* Prognostic index of prognostic model, *MAFLD* Metabolic-associated fatty liver disease, *TyG* Triglyceride and glucose index, *BMI* Body Mass Index, *TG* Triglycerides, *HDL-C* High-density lipoprotein cholesterol, *CI* Confidence interval

**Fig. 4:**
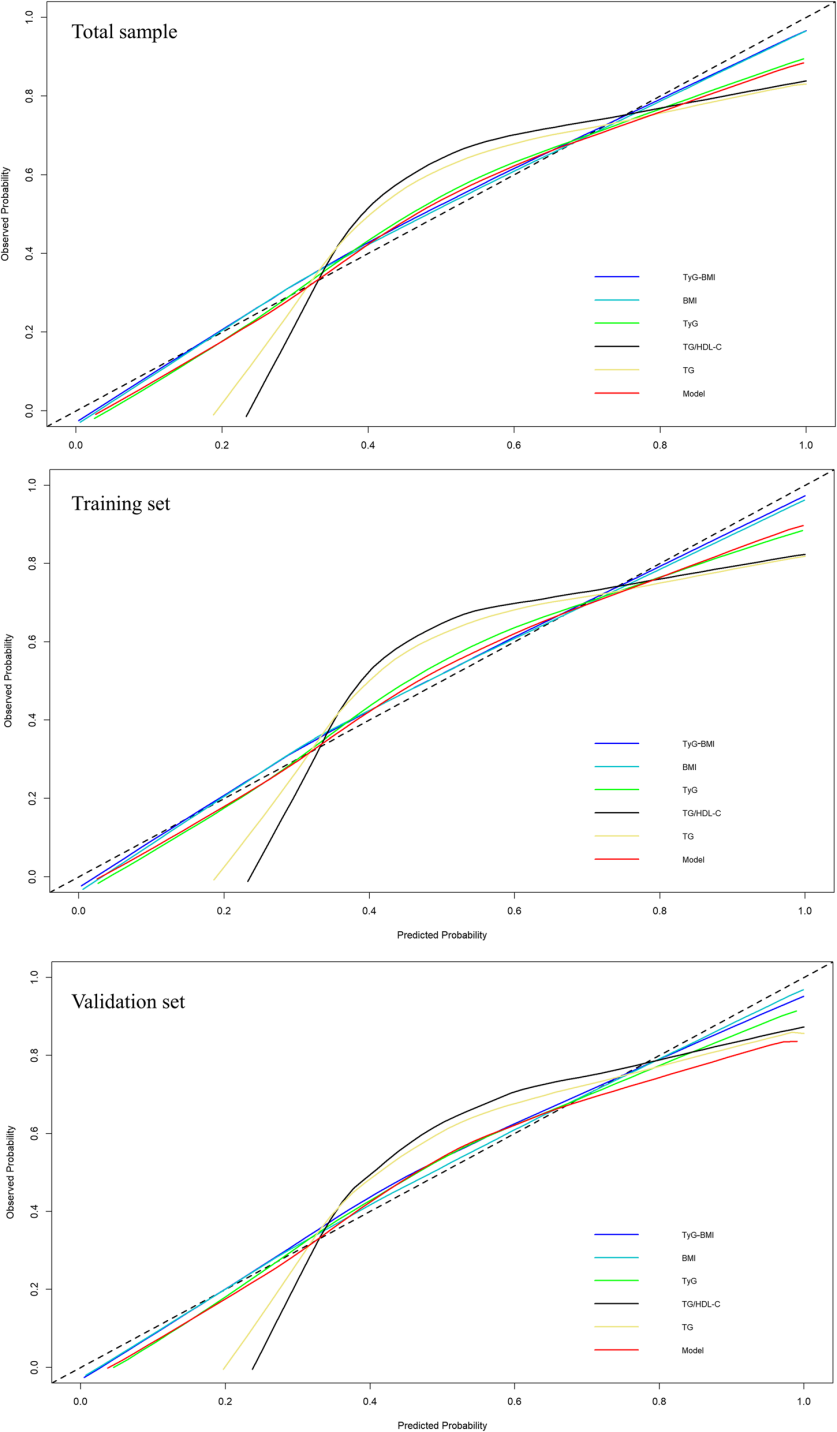
Calibration plot of TyG-BMI, BMI, TyG, TyG/HDL-C, TG, and the Model for MAFLD. Note: The dotted line segment connecting the lower left corner to the upper right corner serves as the reference line. The solid lines in colors are plotted based on the predicted probability of MAFLD and observed probability of MAFLD. Model: prognostic model. Abbreviation: MAFLD: Metabolic-associated fatty liver disease; TyG: triglyceride and glucose index; BMI: Body Mass Index; HDL: high-density lipoprotein cholesterol; TG: triglycerides

**Fig. 5:**
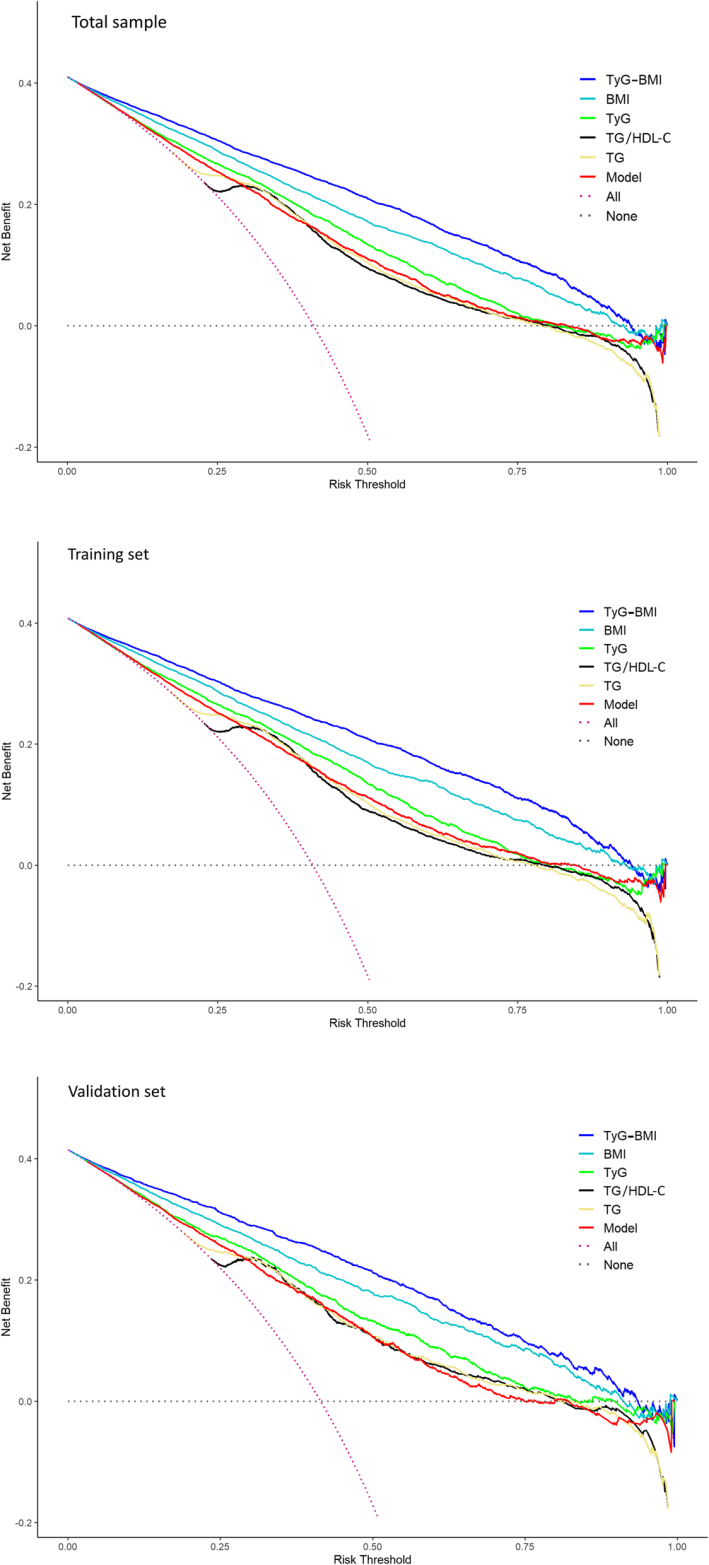
DCA curves of TyG-BMI, BMI, TyG, TyG/HDL-C, TG, and the Model for MAFLD. Note: the horizontal axis represents the risk threshold, and is the reference probability of whether an individual would develop MAFLD. The vertical axis represents the net benefit rate. “None” in the DCA suggested that all participants were non-MAFLD, “All” means that all participants were MAFLD. The lines of each index showed the clinical benefit would be obtained by using this index. Under the same threshold probability, a larger net benefit implied that the individual could obtain a higher benefit using the diagnosis. Model: prognostic model. Abbreviation: MAFLD: Metabolic-associated fatty liver disease; TyG: triglyceride and glucose index; BMI: Body Mass Index; HDL: high-density lipoprotein cholesterol; TG: triglycerides; DCA: Decision curve analysis

The calibration plots were used to compare the predicted probability of MAFLD event (abscissa) with the observed probability of MAFLD event (ordinate) after bias correction (Fig. 4). The TyG-BMI showed the highest consistency between predicted and observed probabilities of MAFLD. Although TG/HDL-C and TG showed high AUCs, they showed deviations from the reference line in the calibration plot. Besides, we employed DCA to compare the diagnostic value for net clinical benefits (Fig. 5). The results showed that the TyG-BMI still had the highest net clinical benefits in predicting MAFLD. The AUCs, sensitivity, specificity, and clinical benefits of each indicator in the training and validation samples were similar to the total sample (Figs. 3, 4 and 5, Table 2).

### Replicability of the findings

After excluding 2,674 participants with at least one missing data, the remaining 5,294 participants without missing data were used for replicability of the findings, and their characteristics were similar to the total participants (Table S1). We found TyG-BMI, BMI, TyG, TG/HDL-C, and TG still ranked top five important predictors (Figure S1), with the TyG-BMI showed highest AUC (0.882) and the most accurate predictor of MAFLD (cut-off value: 218.70, sensitivity: 0.837, specificity: 0.755) (Table S2). The results of comparison of prediction performance among five important predictors and prognostic index in participants without missing data were similar to the results in all participants (Figures S2-S5).

## Discussion

This cross-sectional study, based on a large sample of employed adults, estimated the prediction performance of a set of indicators, including demographic, anthropometric, lifestyle, psychological, and biochemical indicators, in predicting MAFLD. We found that TyG-BMI, BMI, TyG, TG/HDL-C, and TG ranked the top five important indicators. The AUCs of the five indicators were all over 0.7, with TyG-BMI (cut-off value: 218.284, sensitivity: 81.7%, specificity: 78.3%) as the most sensitive and specific indicators for predicting MAFLD. All five variables showed higher prediction performance and net benefit than the prognostic index based on other indicators selected by the multivariate regression model.

It is estimated that over 7% of individuals with MAFLD had developed liver fibrosis [42]. In the current study, over 40% of the participants were living with MAFLD, suggesting that the risk of development of liver fibrosis would be about 2.8%. Early identification of MAFLD with a simple diagnostic index would benefit early intervention and management. We observed five important predictors for MAFLD risk by random forest [17], including TyG-BMI, BMI, TyG, TG/HDL-C and TG. This finding suggested that the predictive ability of biomarkers for MAFLD was generally better than the demographic, anthropometrics, lifestyles, and psychological indicators. Besides, since the previous studies on the predictors for MAFLD were participants recruited from hospitals or general population [17, 20–22, 43, 44], our study can provide a whole picture of predictors for MAFLD among employed adults who were relatively young while lacking attention to their health status.

TyG-BMI was the most powerful predictor for MAFLD, and was superior to other indicators. There are some explanations. First, TyG-BMI is a compositive index including TyG, and they may have similar pathogenesis for MAFLD. Previous studies suggested that both TyG and TyG-BMI were proposed as effective substitutes for IR, which had a crucial role in the development of MAFLD [16, 45]. TyG-BMI was also recommended for investigating IR risk [46], liver fibrosis risk [47], diabetic kidney disease [48] and cardiovascular diseases [49], and may play an important role in predicting other prognoses. Second, the predictive ability of TyG and related parameters can be explained by the two core components (i.e., FPG and TG) which are critical in the development of MAFLD [50]. Glucotoxicity and lipotoxicity mechanisms might exist in the association between TyG/TyG-BMI and MAFLD [15, 51], among which some metabolites associated with insulin resistance were produced and further accounted for the pathogenesis of MAFLD [50]. Third, TyG-BMI might mediate the association between other indicators and MAFLD. As a previous study in Type 2 diabetes (T2DM) patients suggested that TyG-BMI partly mediated the association between γ-Glutamyl transpeptidase (GGT)/HDL and MAFLD incidence [52]. Our findings suggested that TyG-BMI performed better than BMI and TyG to predict MAFLD risk with the highest AUC (0.884). Previous studies reported similar results that the TyG-BMI in diagnosing MAFLD had ROC ranging from 0.675–0.956 [17, 47, 53].

TyG and BMI showed higher prediction performance followed by TyG-BMI, and some advantages deserved attention. First, TyG was effective to screen steatosis, and was superior to other indicators for MAFLD [54]. Besides, the mean TyG (8.86 mmol/L) of participants in our study was higher than the threshold (TyG ≥ 8.5 mmol/L) proposed for identifying MAFLD among the general population in China [25], and was also higher than the mean level among the general population in Korea [55] and Japan [56]. The difference might be explained by the heterogeneity in metabolic status between the employed adults and the general population. Since hypertriglyceridemia increases the transport of free fatty acids to the liver and further leads to fat accumulation, hepatic IR, and glucose output [44], the effect of TyG on MAFLD should be concerned even the index of BMI was absent [57]. The AUC of TyG in our employed adults was 0.795, and the sensitivity and specificity were 0.779 and 0.677, respectively, slightly higher than the results of previous studies among the health examination population in China (AUC = 0.761–0.782, with 70.6%-72.2% sensitivity and 69.1%-70.5% specificity) [25, 58]. Our findings suggested the AUC of BMI is followed by TyG-BMI in predicting MAFLD. As a component of MAFLD, BMI seems reasonable to be used as a tool for diagnosing the risk of MAFLD. It’s estimated that over 65% of individuals with BMI > 40 kg/m^2^ have MAFLD [59]. Caution should be made when taking the BMI as the individual predictor since the existence of lean MAFLD [10].

TG/HDL-C, TG, and prognostic index showed relatively high AUC. TG/HDL-C was a proven predictor of IR [59], and accounted for its association with MAFLD through IR. During the IR-MAFLD pathway, the TG over-enriched LDL particles were also promoted to increase the TG/HDL-C [60] Some prospective evidence suggested that TG/HDL-C was strongly associated with MAFLD in the general population [61]. TG/HDL-C was also reported to be a crucial predictor of cardiovascular diseases [62], highlighting its role in predicting poor prognosis. In addition, the prognostic index based on Nomogram showed an acceptable AUC of 0.763, while it was still lower than the top five important indicators selected by random forest. The possible reason may be that we excluded the five powerful predictors in the synthetic composite indicator. A recent study among general population showed the diagnostic accuracy of Nomogram that contains powerful predictors (e.g., TyG and BMI) for MAFLD was better than other existing models [22]. Our study further suggested that powerful predictors are crucial in predicting MAFLD, which may not be replaced by a synthetic composite indicator excluding these powerful predictors.

The strength of our study is that we focused on employed adults who was rarely understudied and considered comprehensive indicators including demographics, anthropometric, lifestyle, psychological, and biochemical indicators in predicting MAFLD. However, some limitations should be cautious. First, under the “multi-hit” pathogenesis theory of MAFLD [14], there are some crucial determinants of MAFLD that we failed to consider, such as IR, hormones, and gut microbiota. Second, the diagnosis of MAFLD is partly dependent on the examiners. Since we use liver ultrasound rather than the gold-standard of liver biopsy, the detection might have limited sensitivity [63]. Third, as a health check-up, there were no biopsy data to confirm the extent of steatosis and the severity of liver fibrosis. Our model therefore can’t provide information on the likelihood of disease severity and predicting the extent of steatosis and fibrosis. Third, causality may not be perfectly made since this is a cross-sectional study, and longitude studies are needed to determine the accuracy of predictors in predicting incidence of MAFLD. Forth, as the information on plasma insulin levels were not available to define MAFLD in this study, we may have missed on patients with MAFLD that had poor insulin sensitivity but no other metabolic dysfunction. Fifth, our participants were not randomly selected and our findings may not be generalized without caution to the overall employed adults.

## Conclusion

This study compared the prediction performance of a set of demographics, anthropometrics, lifestyle, psychological, and biochemical indicators for MAFLD risk among a large sample of employed adults in China. We found that TyG-BMI showed the highest prediction performance, followed by BMI, TyG, TG/HDL-C, and TG. This study can provide a picture of comprehensive predictors about their ability in predicting the MAFLD risk and aid the informed choice of medication and non-pharmacological interventions for MAFLD management among employed adults.

## Supplementary Information

**Additional file 1: Figure S1.** Importance score of candidate predictors in participants without missing data. Note: variable importance suggested the difference in prediction error when feature values are altered randomly to the prediction error, with a higher difference correspondence to a higher importance of the feature. Abbreviation: TyG: triglyceride and glucose index; BMI: Body Mass Index; TG: triglycerides; HDL-C: high-density lipoprotein cholesterol; ALT: serum alanine transaminase; AST: aspartate transaminase; LDL-C: low-density lipoprotein cholesterol; FPG: fasting plasma glucose; UA: uric acid; TC: total cholesterol; GLB: globulin; DBIL: direct bilirubin; TP: total protein; TBIL: total bilirubin; SII: Systemic immune-inflammation index; CREA: creatinine; IBIL: indirect bilirubin; UREA: urea; ALB: albumin. **Figure S2.** Nomogram of the diagnostic model in participants without missing data. Note: Drawing a vertical line from the axis of each predictor until it reaches the line labeled by “points”, we can calculate the point of each predictor. The summed score of points of all predictors was the total points. Drawing a vertical line in the line labeled “Total Points” from the obtained value of total points until it intercepts the line labeled “Risk”, we can obtain the risk (predicted probability) of MAFLD for a given individual. Abbreviation: MAFLD: Metabolic-associated fatty liver disease; FPG: fasting plasma glucose; CREA: creatinine; SII: Systemic immune-inflammation index; UA: uric acid. Total Points = 2.056*(Age-20)+8.733*Sex+10.941*Marital status+18.516*Work type +11.245*Smoking +100.00*FPG+44.935*CREA+0.026*SII +77.249*UA. **Figure S3.** ROC curves of TyG-BMI, BMI, TyG, TyG/HDL-C, TG, and the Model for MAFLD in participants without missing data. Note: Model: prognostic model. Abbreviation: TyG: triglyceride and glucose index; BMI: Body Mass Index; HDL: high-density lipoprotein cholesterol; TG: triglycerides. **Figure S4.** Calibration plot of TyG-BMI, BMI, TyG, TyG/HDL-C, TG, and the Model for MAFLD in participants without missing data. Note: The dotted line segment connecting the lower left corner to the upper right corner serves as the reference line. The solid lines in colors are plotted based on the predicted probability of MAFLD and observed probability of MAFLD. Model: prognostic model. Abbreviation: TyG: triglyceride and glucose index; BMI: Body Mass Index; HDL: high-density lipoprotein cholesterol; TG: triglycerides. **Figure S5.** DCA curves of TyG-BMI, BMI, TyG, TyG/HDL-C, TG, and the Model for MAFLD in participants without missing data. Note: the horizontal axis represents the risk threshold, and is the reference probability of whether an individual would develop MAFLD. The vertical axis represents the net benefit rate. “None” in the DCA suggested that all participants were non-MAFLD, “All” means that all participants were MAFLD. The lines of each index showed the clinical benefit would be obtained by using this index. Under the same threshold probability, a larger net benefit implied that the individual could obtain a higher benefit using the diagnosis. Model: prognostic model. Abbreviation: TyG: triglyceride and glucose index; BMI: Body Mass Index; HDL: high-density lipoprotein cholesterol; TG: triglycerides; DCA: Decision curve analysis. **Table S1.** The basic characteristics of the participants without missing data. **Table S2.** AUC of TyG-BMI, BMI, TyG, TyG/HDL-C, TG, and the Model in predicting MAFLD risk in participants without missing data.

## Data Availability

The datasets analysed during the current study are not publicly available due to the requirements of each respondent that individual data cannot be disclosed, but datasets are available from the corresponding author on reasonable request.
