# Supplementary material for: Comparison of the prognostic value of a comprehensive set of predictors in identifying risk of metabolic-associated fatty liver disease among employed adults
Source: BMC Public Health. 2023 Mar 29;23:584. doi: 10.1186/s12889-023-15365-9 (PMC10053528; doi:10.1186/s12889-023-15365-9)
Supplement: Supplementary file 1 — Additional file 1: Figure S1. Importance score of candidate predictors in participants without missing data. Note: variable importance suggested the difference in prediction error when feature values are altered randomly to the prediction error, with a higher difference correspondence to a higher importance of the feature. Abbreviation: TyG: triglyceride and glucose index; BMI: Body Mass Index; TG: triglycerides; HDL-C: high-density lipoprotein cholesterol; ALT: serum alanine transaminase; AST: aspartate transaminase; LDL-C: low-density lipoprotein cholesterol; FPG: fasting plasma glucose; UA: uric acid; TC: total cholesterol; GLB: globulin; DBIL: direct bilirubin; TP: total protein; TBIL: total bilirubin; SII: Systemic immune-inflammation index; CREA: creatinine; IBIL: indirect bilirubin; UREA: urea; ALB: albumin. Figure S2. Nomogram of the diagnostic model in participants without missing data. Note: Drawing a vertical line from the axis of each predictor until it reaches the line labeled by “points”, we can calculate the point of each predictor. The summed score of points of all predictors was the total points. Drawing a vertical line in the line labeled “Total Points” from the obtained value of total points until it intercepts the line labeled “Risk”, we can obtain the risk (predicted probability) of MAFLD for a given individual. Abbreviation: MAFLD: Metabolic-associated fatty liver disease; FPG: fasting plasma glucose; CREA: creatinine; SII: Systemic immune-inflammation index; UA: uric acid. Total Points = 2.056*(Age-20)+8.733*Sex+10.941*Marital status+18.516*Work type +11.245*Smoking +100.00*FPG+44.935*CREA+0.026*SII +77.249*UA. Figure S3. ROC curves of TyG-BMI, BMI, TyG, TyG/HDL-C, TG, and the Model for MAFLD in participants without missing data. Note: Model: prognostic model. Abbreviation: TyG: triglyceride and glucose index; BMI: Body Mass Index; HDL: high-density lipoprotein cholesterol; TG: triglycerides. Figure S4. Calibration plot of TyG-BMI, BMI, TyG, T [file 12889_2023_15365_MOESM1_ESM.docx]

**Supplementary Materials**

Comparison of the prognostic value of a comprehensive set of predictors in identifying risk of metabolic-associated fatty liver disease among employed adults


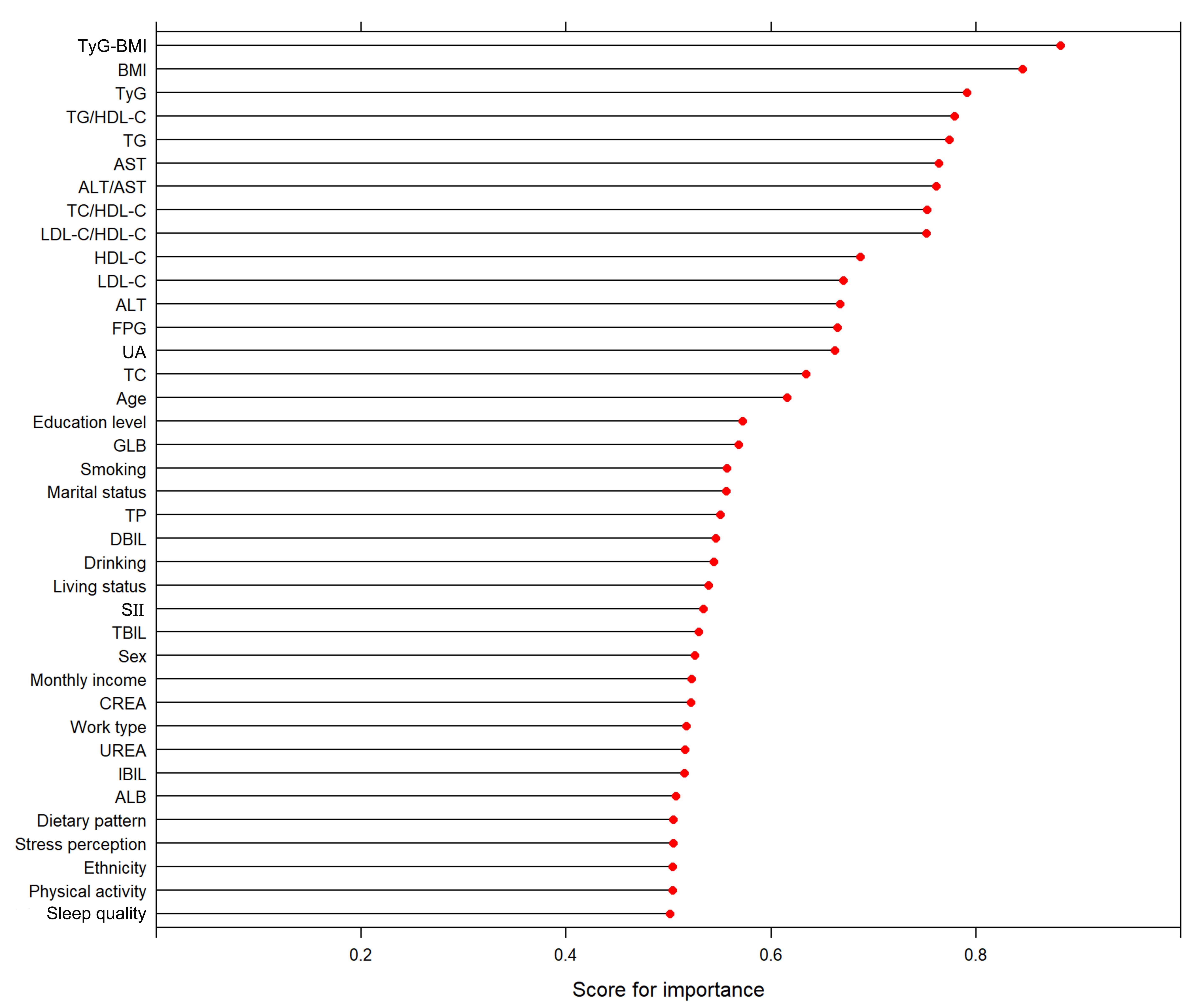


**Figure S1.** Importance score of candidate predictors in participants without missing data

Note: variable importance suggested the difference in prediction error when feature values are altered randomly to the prediction error, with a higher difference correspondence to a higher importance of the feature.

Abbreviation: TyG: triglyceride and glucose index; BMI: Body Mass Index; TG: triglycerides; HDL-C: high-density lipoprotein cholesterol; ALT: serum alanine transaminase; AST: aspartate transaminase; LDL-C: low-density lipoprotein cholesterol; FPG: fasting plasma glucose; UA: uric acid; TC: total cholesterol; GLB: globulin; DBIL: direct bilirubin; TP: total protein; TBIL: total bilirubin; SⅡ: Systemic immune-inflammation index; CREA: creatinine; IBIL: indirect bilirubin; UREA: urea; ALB: albumin.


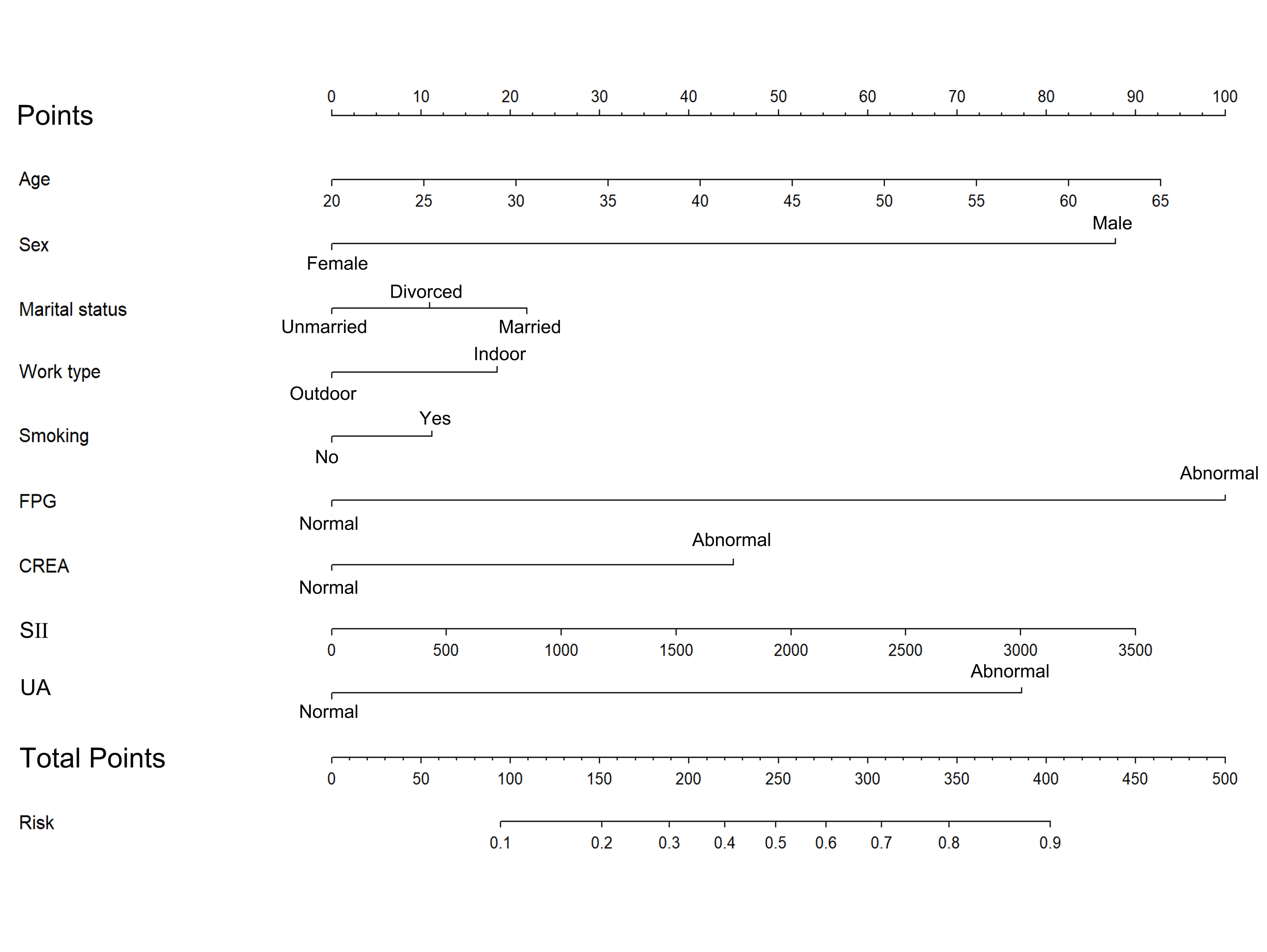


**Figure S2.** Nomogram of the diagnostic model in participants without missing data

Note: Drawing a vertical line from the axis of each predictor until it reaches the line labeled by “points”, we can calculate the point of each predictor. The summed score of points of all predictors was the total points. Drawing a vertical line in the line labeled “Total Points” from the obtained value of total points until it intercepts the line labeled “Risk”, we can obtain the risk (predicted probability) of MAFLD for a given individual.

Abbreviation: MAFLD: Metabolic-associated fatty liver disease; FPG: fasting plasma glucose; CREA: creatinine; SⅡ: Systemic immune-inflammation index; UA: uric acid. Total Points=2.056*(Age-20)+8.733*Sex+10.941*Marital status+18.516*Work type +11.245*Smoking +100.00*FPG+44.935*CREA+0.026*SⅡ +77.249*UA


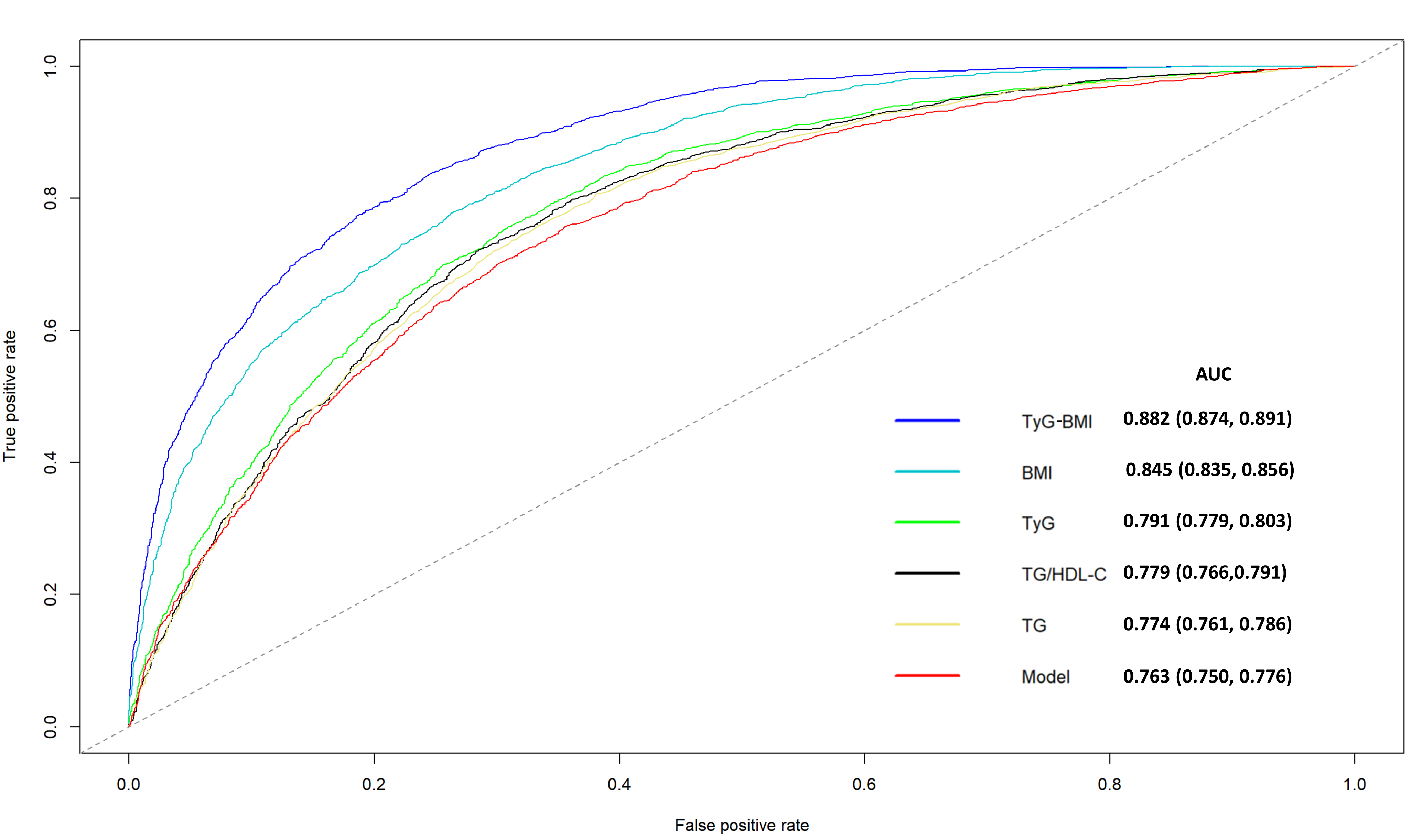


**Figure S3.** ROC curves of TyG-BMI, BMI, TyG, TyG/HDL-C, TG, and the Model for MAFLD in participants without missing data

Note: Model: prognostic model. Abbreviation: MAFLD: Metabolic-associated fatty liver disease; TyG: triglyceride and glucose index; BMI: Body Mass Index; HDL: high-density lipoprotein cholesterol; TG: triglycerides.


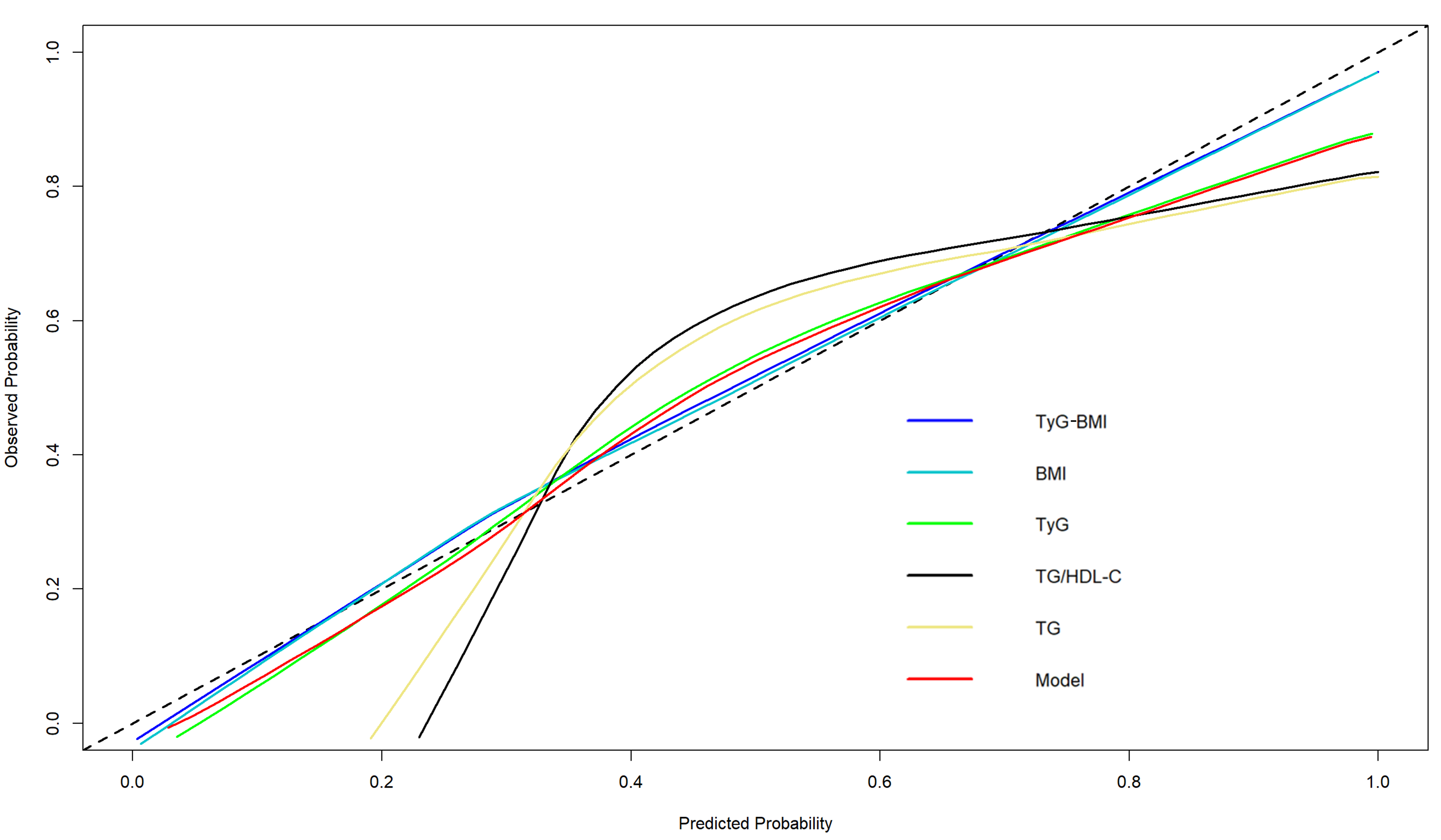


**Figure S4.** Calibration plot of TyG-BMI, BMI, TyG, TyG/HDL-C, TG, and the Model for MAFLD in participants without missing data

Note: The dotted line segment connecting the lower left corner to the upper right corner serves as the reference line. The solid lines in colors are plotted based on the predicted probability of MAFLD and observed probability of MAFLD.

Model: prognostic model. Abbreviation: MAFLD: Metabolic-associated fatty liver disease; TyG: triglyceride and glucose index; BMI: Body Mass Index; HDL: high-density lipoprotein cholesterol; TG: triglycerides.


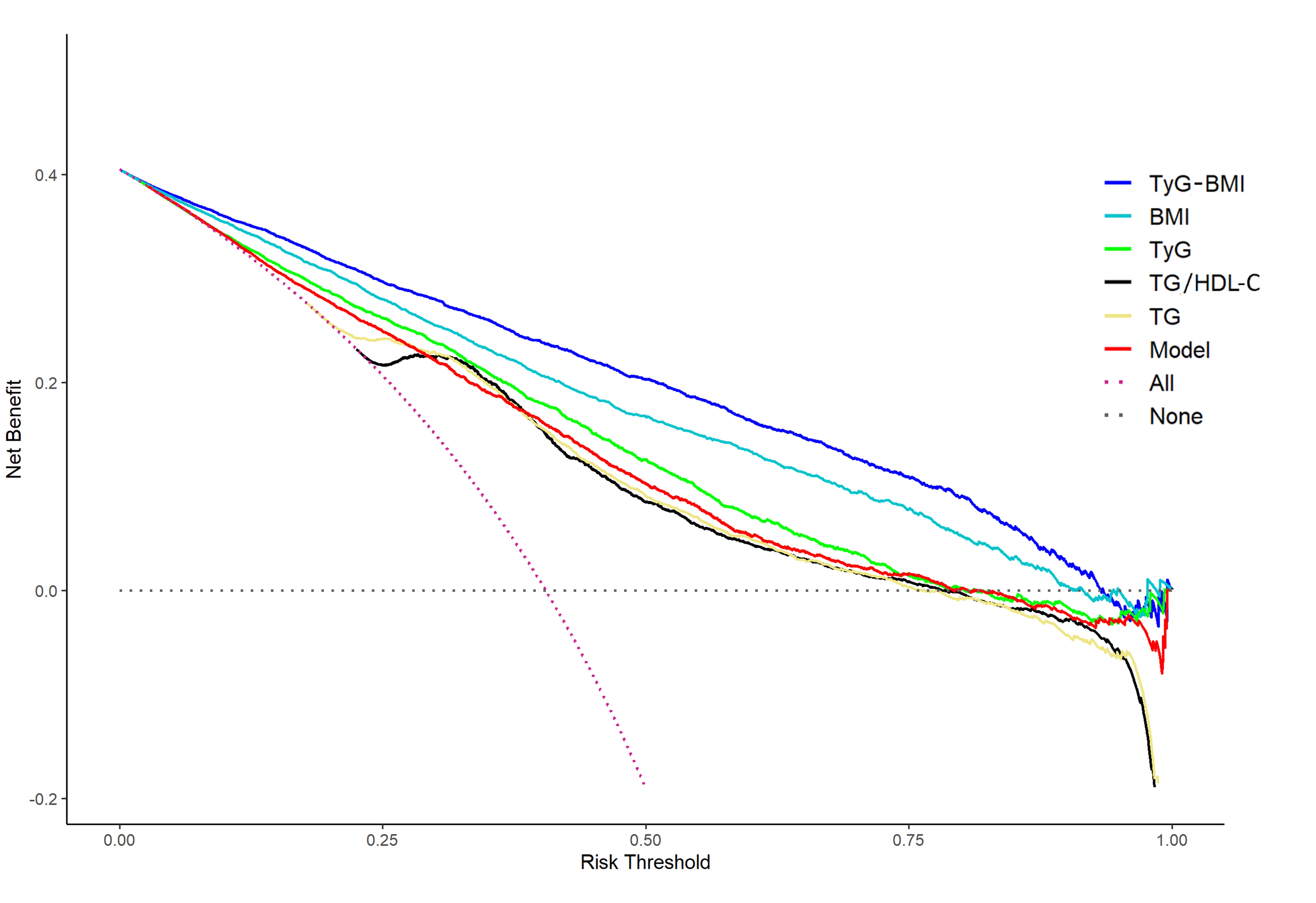


**Figure S5.** DCA curves of TyG-BMI, BMI, TyG, TyG/HDL-C, TG, and the Model for MAFLD in participants without missing data

Note: the horizontal axis represents the risk threshold and is the reference probability of whether an individual would develop MAFLD. The vertical axis represents the net benefit rate. “None” in the DCA suggested that all participants were non-MAFLD, “All” means that all participants were MAFLD. The lines of each index showed the clinical benefit would be obtained by using this index. Under the same threshold probability, a larger net benefit implied that the individual could obtain a higher benefit using the diagnosis.

Model: prognostic model. Abbreviation: MAFLD: Metabolic-associated fatty liver disease; TyG: triglyceride and glucose index; BMI: Body Mass Index; HDL: high-density lipoprotein cholesterol; TG: triglycerides; DCA: Decision curve analysis.

**Table S1.** The basic characteristics of the participants without missing data

| Variable | Total | MAFLD (n[%] or mean±SD) | | |
| --- | --- | --- | --- | --- |
|  |  | No | Yes | P value |
|  | N=5,294 | N=3,150 | N=2,144 |  |
| Sex |  |  |  | <0.001 |
| Female | 227 (4.29) | 200 (6.35) | 27 (1.26) |  |
| Male | 5,067 (95.71) | 2,950 (93.65) | 2,117 (98.74) |  |
| Age (years) | 39.37±10.96 | 37.58±11.05 | 42.01±10.26 | <0.001 |
| Ethnicity |  |  |  | 0.293 |
| Han | 4,919 (92.92) | 2,937 (93.24) | 1,982 (92.44) |  |
| Minority | 375 (7.08) | 213 (6.76) | 162 (7.56) |  |
| Educational level |  |  |  | <0.001 |
| High school and below | 2,198 (41.52) | 1,123 (35.65) | 1,075 (50.14) |  |
| College degree or above | 3,096 (58.48) | 2,027 (64.35) | 1,069 (49.86) |  |
| Marital status |  |  |  | <0.001 |
| Married/cohabitation | 3,760 (71.02) | 2,084 (66.16) | 1,676 (78.17) |  |
| Unmarried | 1,287 (24.31) | 930 (29.52) | 357 (16.65) |  |
| Separated/Divorced/Widowed | 247 (4.67) | 136 (4.32) | 111 (5.18) |  |
| Monthly household income (*yuan*) | |  |  | 0.001 |
| <6,000 | 1,593 (30.09) | 1,005 (31.90) | 588 (27.43) |  |
| ≥6,000 | 3,701 (69.91) | 2,145 (68.10) | 1,556 (72.57) |  |
| Work type |  |  |  | 0.735 |
| Work outdoor | 951 (17.96) | 571 (18.13) | 380 (17.72) |  |
| Work indoor | 4,343 (82.04) | 2,579 (81.87) | 1,764 (82.28) |  |
| Living status |  |  |  | <0.001 |
| Living with family | 3,284 (62.03) | 1,845 (58.57) | 1,439 (67.12) |  |
| Living with colleagues or friends | 899 (16.98) | 609 (19.33) | 290 (13.53) |  |
| Living with strangers | 172 (3.25) | 109 (3.46) | 63 (2.94) |  |
| Living alone | 939 (17.74) | 587 (18.63) | 352 (16.42) |  |
| BMI (kg/m^2^) | 24.52 (3.56) | 22.85 (2.81) | 26.98 (3.10) | <0.001 |
| Drinking |  |  |  | <0.001 |
| Non-drinker | 1,809 (34.17) | 1,150 (36.51) | 659 (30.74) |  |
| Ex-drinker | 235 (4.44) | 133 (4.22) | 102 (4.76) |  |
| Occasional drinker | 2,224 (42.01) | 1,343 (42.63) | 881 (41.09) |  |
| Current drinker | 1,026 (19.38) | 524 (16.63) | 502 (23.41) |  |
| Smoking |  |  |  | <0.001 |
| Non-smoker | 1,875 (35.42) | 1,252 (39.75) | 623 (29.06) |  |
| Ex-smoker | 390 (7.37) | 196 (6.22) | 194 (9.05) |  |
| Occasional smoker | 628 (11.86) | 391 (12.41) | 237 (11.05) |  |
| Current smoker | 2,401 (45.35) | 1,311 (41.62) | 1,090 (50.84) |  |
| Dietary pattern |  |  |  | 0.814 |
| Ideal dietary pattern | 1,817 (34.32) | 1,092 (34.67) | 725 (33.82) |  |
| Medium dietary pattern | 3,239 (61.18) | 1,917 (60.86) | 1,322 (61.66) |  |
| Poor dietary pattern | 238 (4.50) | 141 (4.48) | 97 (4.52) |  |
| Physical activity (MET-min/w) | |  |  | 0.616 |
| <600 | 1,773 (33.49) | 1,055 (33.49) | 718 (33.49) |  |
| 600-2,999 | 1,786 (33.74) | 1,077 (34.19) | 709 (33.07) |  |
| ≥3,000 | 1,735 (32.77) | 1,018 (32.32) | 717 (33.44) |  |
| Sleep quality |  |  |  | 0.594 |
| Good | 2,684 (50.70) | 1,587 (50.38) | 1,097 (51.17) |  |
| Poor | 2,610 (49.30) | 1,563 (49.62) | 1,047 (48.83) |  |
| Stress perception | 16.33 (3.80) | 16.33 (3.84) | 16.34 (3.74) | 0.904 |
| TBIL (μmold/L) | 16.48±6.21 | 16.70±6.17 | 16.16±6.25 | 0.002 |
| DBIL (μmold/L) | 5.34±2.32 | 5.48±2.31 | 5.13±2.33 | <0.001 |
| IBIL (μmold/L) | 11.14±4.21 | 11.22±4.16 | 11.02±4.27 | 0.096 |
| ALT (IU/L) | 23.35±11.96 | 21.07±9.44 | 26.69±14.28 | <0.001 |
| AST (IU/L) | 29.81±23.84 | 22.84±17.07 | 40.04±28.28 | <0.001 |
| TP (g/L) | 75.77±4.37 | 75.42±4.00 | 76.29±4.82 | <0.001 |
| ALB (g/L) | 45.38±2.36 | 45.40±2.37 | 45.35±2.35 | 0.499 |
| GLB (g/L) | 30.39±3.74 | 30.03±3.33 | 30.93±4.22 | <0.001 |
| FPG (mmol/L) | 5.51±1.34 | 5.24±0.94 | 5.90±1.70 | <0.001 |
| UA (μmold/L) | 398.04±86.87 | 378.35±79.98 | 426.96±88.51 | <0.001 |
| UREA (mmol/L) | 4.85±1.19 | 4.82±1.19 | 4.88±1.19 | 0.069 |
| CREA (mmol/L) | 72.28±12.25 | 71.79±11.80 | 72.99±12.84 | <0.001 |
| TG (mmol/L) | 2.07±1.86 | 1.57±1.24 | 2.81±2.32 | <0.001 |
| TC (mmol/L) | 4.68±0.90 | 4.51±0.84 | 4.93±0.92 | <0.001 |
| HDL-C (mmol/L) | 1.35±0.30 | 1.42±0.30 | 1.24±0.26 | <0.001 |
| LDL-C (mmol/L) | 2.75±0.67 | 2.60±0.64 | 2.98±0.66 | <0.001 |
| TyG | 8.88±0.68 | 8.62±0.55 | 9.27±0.65 | <0.001 |
| ALT/AST | 0.95±0.45 | 1.08±0.50 | 0.77±0.28 | <0.001 |
| LDL-C/HDL-C | 2.16±0.89 | 1.91±0.64 | 2.52±1.06 | <0.001 |
| TG/HDL-C | 1.88±4.91 | 1.25±1.58 | 2.80±7.38 | <0.001 |
| TC/HDL-C | 3.65±1.46 | 3.29±0.91 | 4.18±1.90 | <0.001 |
| TyG-BMI | 218.73±41.24 | 197.36±30.54 | 250.12±34.25 | <0.001 |
| SⅡ | 444.81±223.39 | 437.84±227.34 | 455.05±217.11 | 0.006 |

Note: MAFLD: metabolic-associated fatty liver disease; BMI: Body Mass Index; MET: Metabolic Equivalent of Task; TBIL: total bilirubin; DBIL: direct bilirubin; IBIL: indirect bilirubin; ALT:serum alanine transaminase; AST: aspartate transaminase; TP: total protein; ALB: albumin; GLB: globulin; FPG: fasting plasma glucose; UA: uric acid; UREA: urea; CREA: creatinine; TG: triglycerides; TC: total cholesterol; HDL-C: high-density lipoprotein cholesterol; LDL-C: low-density lipoprotein cholesterol; TyG: triglyceride and glucose index; SⅡ: Systemic immune-inflammation index; SD: standard deviation.

**Table S2.** AUC of TyG-BMI, BMI, TyG, TyG/HDL-C, TG, and the Model in predicting MAFLD risk in participants without missing data

| **Variable** | **AUC** | **95%CI** | **Cut-off value** | **Sensitivity (%)** | **Specificity (%)** | **Youden's index** |
| --- | --- | --- | --- | --- | --- | --- |
| TyG-BMI | 0.882 | (0.874, 0.891) | 218.170 | 0.837 | 0.755 | 0.592 |
| BMI | 0.845 | (0.835, 0.856) | 24.575 | 0.778 | 0.737 | 0.515 |
| TyG | 0.791 | (0.779, 0.803) | 8.779 | 0.792 | 0.656 | 0.448 |
| TG/HDL-C | 0.779 | (0.766, 0.791) | 1.115 | 0.799 | 0.639 | 0.437 |
| TG | 0.774 | (0.761, 0.786) | 1.475 | 0.806 | 0.619 | 0.425 |
| Model | 0.763 | (0.750, 0.776) | -0.562 | 0.757 | 0.644 | 0.402 |

Note: Model: prognostic model. Abbreviation: AUC: area under the receiver operating characteristic curve; MAFLD: metabolic-associated fatty liver disease; TyG: triglyceride and glucose index; BMI: Body Mass Index; TG: triglycerides; HDL-C: high-density lipoprotein cholesterol. CI: confidence interval.
